# Study of Psychosocial and Academic Indicators in Young Adults from Andalucía, Spain

**DOI:** 10.3390/ijerph18020363

**Published:** 2021-01-06

**Authors:** Tamara Espejo-Garcés, Javier Cachón-Zagalaz, Félix Zurita-Ortega, Gabriel González-Valero, José Luis Ubago-Jiménez

**Affiliations:** 1 Research Group HUM-653, Faculty of Education Sciences, University of Jaén, 23008 Jaén, Spain; tamaraeg@correo.ugr.es; 2 Department of Didactics of Musical, Plastic and Corporal Expression, University of Jaén, 23008 Jaén, Spain; jcachon@ujaen.es; 3 Department of Didactics of Musical, Plastic and Corporal Expression, University of Granada, 18071 Granada, Spain; felixzo@ugr.es (F.Z.-O.); jlubago@ugr.es (J.L.U.-J.)

**Keywords:** self-concept, emotional intelligence, harmful substances, academic stress, university, physical education

## Abstract

Background: The university years bring a great deal of vital changes. In addition, psycho-social factors play a key role in university students’ development and their consumption of harmful substances. The aim is to analyse academic performance according to psychosocial factors, self-concept, emotional intelligence and harmful substance consumption in a sample of future Physical Education teachers. Methods: The present study was carried out with a sample of 775 undergraduate students who were studying for the Primary Education Degree in Physical Education in Andalusia, Spain. The main instruments used include an ad hoc questionnaire, the Self-Concept Form-5 test, Alcohol Use Disorders Identification Test, Fagerström Test for Nicotine Dependence and the Emotional Intelligence Inventory adapted to Sport. Results: The results show that young people who have a greater general self-concept and self-emotional management are those who access the university degree through Vocational Training. In relation to the average record mark, it was observed that participants with marks of notable and outstanding obtained higher scores in general self-concept, academic dimension and hetero-emotional management. Those with grades of passed and outstanding were those with higher levels in the physical dimension of self-concept and emotional use. In relation to receiving a scholarship to study, it has been shown that those university students who do perceive it have higher levels in most of the dimensions of emotional intelligence and self-concept. Conclusions: Finally, the harmful substances did not show significant relationships with academic factors, except between tobacco and the average mark of the file.

## 1. Introduction

The university stage is a time period where young people start higher education, mostly to obtain professional qualifications that allow them to be successfully introduced into the world of work [1,2]. During this period, many changes take place in their daily lives, customs and relationships that modify and affect their identity [3,4], as well as psychosocial processes such as academic attrition and stress, the management of emotions when faced with difficult situations, the development of non-healthy behaviours such as the lack of physical activity and, therefore, sedentary lifestyle, or the consumption of harmful substances [5,6].

In this regard, self-concept is a psychosocial construct that helps to shape the personality, thereby changing depending on the individual’s perception of his or her environment, their feelings and thoughts, learning and experiences, and their own and other people’s assessments of their relationship with others [7,8]. The balanced and positive development of self-concept will have an impact on a proper psychological adjustment and the achievement of personal life goals [9]. Likewise, according to Ingles et al. [10], the academic dimension of self-concept can be considered as a predictive factor of behaviour and profiles in the educational context, since those with higher academic self-concept get positive assessments from their peers.

Similarly, emotional intelligence is related to important psychological benefits, since emotional control and management enables people to face different life changes successfully and reduces mental risks and imbalances [11,12]. As stated by Rieffe et al. [13] and Villanueva and Górriz [14], emotional awareness not only involves attention-related processes, but also the identification and differentiation of emotions, the localisation and analysis of changes produced by one’s own and other people’s emotional experiences.

It is considered fundamental that these two constructs are positive among their dimensions, since high levels of self-concept allow an awareness of oneself and of the perceptions about different aspects that converge in the personal and social process of the individual, it will motivate the promotion and treatment of an adequate psychosocial adjustment, where the perception, management and use of emotions result in skills and abilities related to psychological well-being [15]. These positive relationships are the object of study in various research projects, where the idea that having a positive perception of oneself, as well as feeling valid and capable, allows for the creation of a more favourable scenario to develop adequately in various areas [16,17,18,19].

However, Ibarrola [20] indicates that the current trend is to attribute low academic performance to emotional problems and not so much to the capacity of the students. To this end, Broc-Cavero [21] explains the possibility of an overestimation of emotional intelligence by educational policies and the media, due to the presence of various investigations where no significance or low strength relations between emotional intelligence and academic performance can be found [22,23,24,25].

On the other hand, according to the World Health Organization (WHO) [26], the consumption of alcohol and tobacco, among other substances, is one of the main problems related to adolescents’ and young people’s health, causing problems for the full development and growth potential of individuals. Some studies indicate links between alcohol consumption or alcohol dependency, with school absenteeism, mistrust, low academic performance, emotional distress, poor or non-existent expectations, closeness to other drugs consumption, dissatisfaction with life and an increase in family problems, among others [27,28,29].

The need for control, monitoring and treatment of this type of harmful substances is unavoidable, as well as the elements of risk (family and peer group), socio-cultural environment, with a greater focus on adolescents and young people. These stages are considered vulnerable, as these subjects do not usually attach importance to the long-term effects on their health, and these consumptions are found to be accepted and normal social activities by their environment [30,31,32,33].

The contribution of García-Do Nascimiento, Molerio-Pérez and Pedraza [34] is relevant, where they point out the importance that educational contexts have for the development of more adult, healthy and competent personalities, as well as emotional competencies, independence and trust in students.

Based on the above, the main objectives of this study are to describe and analyse the relationship between university access, the average mark in the exam and the acquisition of scholarships with self-concept, emotional intelligence and consumption of harmful substances among university students who are studying for the degree in Physical Education in Primary Education in Andalusia.

## 2. Materials and Methods

### 2.1. Participants and Design

A non-experimental, descriptive, cross-sectional design study was developed using a one-time measure. The sample is established with a total of 775 university students who are studying Physical Education in different provinces of Andalusia (Spain). The representation by sex is 58.7% (*n* = 455) for men and 43.1% (*n* = 320) for women, with an age range between 20 and 53 years (22.45 ± 2.90). Participants were selected by random sampling for convenience. The inclusion criterion used was that they should be in their last academic year and should belong to a Physical Education degree, regardless of the age of the individuals. A representative sample of 775 students of Primary Education Degree Specialising in Physical Education was established, assuming that 67.86% of the universe was sampled (1142), with a total sampling error of 0.02. Therefore, it is considered that the sample adjusts properly to the statistical criteria to ensure its representativeness.

### 2.2. Instruments

Ad-Hoc Questionnaire: A questionnaire designed to record academic aspects such as access to the university, average mark in the academic career and grant award.

Self-concept: Self-Concept Form-5 (AF-5) questionnaire. This instrument was developed by García and Musitu [35] and is based on the multidimensional theoretical model [36,37]. It is composed of 30 items, which are scored using a 5-option Likert-type scale, where 1 is “Never” and 5 is “Always”, the self-concept construct in its social aspect (items 2, 7, 12, 17, 22 and 27), academic/professional (items 1, 6, 11, 16, 21 and 26), emotional (items 3, 8, 13, 18, 23 and 28), family (items 4, 9, 14, 19, 24 and 29) and physical (items 5, 10, 15, 20, 25 and 30). According to the theoretical foundation provided by the writers, a reliability was established using the Cronbach alpha coefficient of α = 0.810, a similar value to the one expressed in this study (α = 0.78).

Scale of alcohol consumption: A questionnaire extracted from the Alcohol Use Disorders Identification Test (AUDIT), elaborated by Saunders et al. [38] and translated into Spanish by Rubio et al. [39]. It is made up of 10 items, eight of them using a five-option Likert type scale, and three applying a three-option Likert type scale, scoring between 0 and 40 points, where 0 to 8 is considered a “non-risk drinker”, 16 to 19 “medium-risk drinker” and 20 or more “high-risk drinker”. For this instrument, an internal consistency of α = 0.77 was obtained.

Tobacco consumption test: Scale extracted from Fagerström Test for Nicotine Dependence (FTND) created by Heatherton, Kozlowski and Frecker [40] and translated into Spanish by Becoña and Vázquez [41]. It consists of 6 items, four of which are dichotomous, and two items are answered by a four-option Likert scale. Thus, after the sum of the scores, 1 and 2 are categorized as “low nicotine dependence”, 3 and 4 as “low to moderate dependence” and between 5 and 7 as “moderately dependent”. The reliability obtained in this instrument has been of α = 0.97.

Questionnaire on Emotional Intelligence adapted to Sport (IED): Adapted from the original instrument by Shutte et al. [42] by García-Coll et al. [43]. It presents four dimensions: emotional perception (items 2, 3, 10, 12, 14, 21, 23 and 28), self-emotional management (items 5, 9, 15, 18, 19, 22, 25 and 29), hetero-emotional management (items 1, 4, 6, 8, 11, 13, 16, 24, 26 and 30) and emotional use (items 7, 17, 20 and 27). It is assessed using a five-point Likert-type scale, where 1 = “Totally disagree” and 5 = “Totally agree”. For this instrument, an internal consistency of α = 0.91 was obtained.

### 2.3. Procedure

Firstly, the participants were asked for their collaboration through a letter drawn up by the Corporal Area of the University of Granada with the nature and purpose of the study, as well as their informed consent to participate in the research. This letter was provided to the university students specializing in Physical Education from the Primary Education Degree in the different provinces of Andalusia through the teachers of the Didactic Department of Musical, Plastic and Corporal Expression and Physical and Sports Education.

The data collection was carried out subsequently, where the instruments were applied during school hours in the different universities without any incidents. The anonymity of all participants was guaranteed. Participants volunteered to participate according to the Declaration of Helsinki in research projects. Finally, it should be noted that a total of 64 questionnaires were discarded due to non-compliance.

### 2.4. Data Analysis

The statistical analysis was carried out with IBM SPSS^®^ 22.0 software (IBM, Armonk, NY, USA). Means, standard deviation and percentages are used for analysing the descriptives, while Student’s T, one-factor ANOVA and contingency tables were used to study associations between variables, depending on the nature of the variables, using the Pearson Chi-Square statistic to determine significance (*p* ≤ 0.05). For the ANOVA analysis, Schaffel’s post hoc test was used to compare groups of academic parameters. The internal reliability of the instruments used was measured using the Cronbach alpha coefficient, setting the Reliability Index at 95.5%.

## 3. Results

### Descriptives

Table 1 shows the distribution by sex, where 58.7% (*n* = 455) are men and 41.3% (*n* = 320) are women, aged from 20 to 53 years (M = 22.45 years; SD = 2.90). Descriptive data are also collected regarding the access to university, average mark, grant awarding, levels of self-concept, emotional intelligence and consumption of harmful substances of university students who are studying for the Primary Education Degree specialising in Physical Education in Andalusia. Regarding academic aspects, it is noted that the average mark of the students is between 5.1 and 10 (M = 7.49; SD = 0.69), with 82.3% (*n* = 638) of students with a mark of notable, 15.6% (*n* = 121) of pass, and with less representation, 2.1% (*n* = 16) with a mark of excellent. As regards access to university, it is established that 88.9% (*n* = 689) of the cases were through the baccalaureate, and 11.1% (*n* = 86) through professional training. It is also stated that those who received a scholarship were 59.7% (*n* = 463), while 40.3% (*n* = 312) did not receive one.

Taking into account the levels of self-concept and its dimensions, the participants show higher average values in physical self-concept (M = 3.77; SD = 0.62) and academic self-concept (M = 3.76; SD = 0.52), with lower values in the general self-concept (M = 3.58; SD = 0.31), the family self-concept (M = 3.56; SD = 0.33) and the social self-concept (M = 3.54; SD. = 0.36). Finally, the emotional dimension stands out with a lower value, with an average value of 3.26 (SD = 0.78).

The results of the analysis of alcohol and tobacco consumption show that 82.2% (*n* = 637) of the participants do not present risk in their relationship with alcohol, as well as 81.7% (*n* = 633) do not smoke; 16.3% (*n* = 126) are classified as low risk drinkers, and 0.9% (*n* = 7) have low tobacco dependency; similar values are inversely found in the medium risk drinker category with 1% (*n* = 8) of the sample, and medium tobacco dependency with 15.5% (*n* = 120). Finally, the values are minimal in relation to those with high-risk consumption, with 0.5% (*n* = 4) in the case of alcohol and 1.9% (*n* = 15) of participants with high tobacco dependence.

Finally, in terms of emotional intelligence and its dimensions, participants present higher average values in emotional perception (M = 4.11; SD = 0.50) and hetero-emotional management (M = 3.99; SD = 0.48), indicating relatively less value for emotional use (M = 3.96; SD = 0.48) and general emotional intelligence (M = 3.95; SD = 0.43). Finally, the dimension of self-emotional management is highlighted with a lower value, with an average value of 3.73 (SD = 0.52), as shown in the following table.

In the study of the analysis between academic aspects and psychosocial factors, no significant differences were established (*p* > 0.05) in terms of the dimensions of self-concept according to the access to university, except with the general self-concept where differences were found between those who access through baccalaureate and those who access through professional training (*p* = 0.041 *), with a higher level of general self-concept for those who access through professional training (M = 3.64) compared to those who access through the baccalaureate (M = 3.57). Similarly, between alcohol and tobacco consumption and university access, there were no statistical differences (*p* > 0.05) between the different parameters. In the study of emotional intelligence with this academic factor, only significant differences were established in the dimension of self-emotional management (*p* ≤ 0.05*), where it is stated that those with higher average levels of self-emotional management are those who accessed through professional training (M = 3.88) compared to those who accessed through the baccalaureate (M = 3.71), as shown in Table 2.

Table 3 indicates the levels of self-concept, harmful substances and emotional intelligence based on the average mark of the students. Significant differences are established (*p* ≤ 0.05 *) between the general self-concept on two of its dimensions (academic and physical) with the average mark, where those who obtain an average mark of notable (M = 3.60) present a greater general self-concept, followed by those who obtain excellent average marks (M = 3.53), and lower average marks for those who pass (M = 3.48). In the academic self-concept, the differences between groups are more remarkable, where those who present higher levels in this dimension are those with the mark of excellent (M = 4.07), followed by those with the mark of notable (M = 3.81) and, finally, with lower values those with the mark of pass (M = 3.43). Regarding the physical self-concept, there are significant scores reported, with those with an average mark of “notable” with higher levels in this dimension (M = 3.81), followed by those with a grade of “passed” (M = 3.63) and, with the lowest levels, those of “excellent” (M = 3.40). In the dimensions of social, family and emotional self-concept, no significant associations were determined (*p* > 0.05).

Regarding the relationship between alcohol and tobacco consumption with the average mark, statistical differences were obtained in the case of tobacco (*p* = 0.013 *), in contrast to the relationship with alcohol consumption (*p* > 0.05). With regard to tobacco consumption, the data show that those who do not smoke in 83.4% (*n* = 528) have a notable mark, in 14.1% (*n* = 89) average mark pass, and with the lowest representation those of excellent in 2.5% (*n* = 16). In the low- and high-dependency categories, the distribution is distributed among those with notable and pass marks, with similar data around 60 and 40%, respectively. It was found that those with an average dependency of 80.8% (*n* = 97) present an average mark of “notable”, with less representation of those with a qualification of “notable” (19.2%) and none of those of “excellent”.

Finally, regarding the levels of emotional intelligence, only in the dimension of hetero-emotional management and emotional use significant differences were established (*p* ≤ 0.05 *) with the average mark. In this way, it was found that those who have higher average levels in hetero-emotional management are those who have an average mark of notable (M = 4.01), followed by those who have an average of excellent (M = 3.93) and, finally, those who have an average mark of pass (M = 3.89). In the dimension of emotional use, those who have an average mark of “notable” are also those who present a higher average value (M = 3.98), while those whose average mark is “passed” have a slightly lower average in this dimension (M = 3.86), and those whose mark is “excellent” are even more so (M = 3.76). No significant associations were obtained in the rest of the dimensions (*p* > 0.05), as can be seen in the following table.

Finally, the analysis of the psychosocial factors according to receiving scholarships is assessed (Table 4). With regard to self-concept and its dimensions, differences were established (*p* ≤ 0.05 *) between the groups of those who did and did not receive a scholarship, in the general self-concept and in three of its dimensions (academic, family and physical). In this regard, the average general self-concept is higher in those who did receive a scholarship (M = 3.60) compared to those who did not (M = 3.55); the same applies to the academic and physical dimensions, with those who received a scholarship showing higher levels (M = 3.82). On the other hand, in the case of the family dimension, those who did not receive a scholarship report higher levels (M = 3.61), while those who did receive a scholarship have lower average levels of family self-concept (M = 3.53).

Regarding the analysis of alcohol and tobacco consumption with the reception of a scholarship, no statistical differences (*p* > 0.05) were obtained between the different parameters. For the levels of participants’ emotional intelligence, only the dimension of emotional perception showed significant associations, where those participants who received a scholarship had higher average levels of emotional perception (M = 4.14) compared to those who did not (M = 4.06).

## 4. Discussion

The present study attempts to define the impact of psychosocial factors according to the academic aspects found among Andalusian university students who are studying for their Primary Education Degree with the specialism in Physical Education. There are several studies that address these constructs in isolation or maintain a similar nature of study [3,6,10,19]. However, to the knowledge of the authors, this is one of the few studies that focus on the specific group of university students of Physical Education at a national level. The relationships between psychosocial and physical-health variables essential to the professional development of physical education teachers were explored. These relationships make it possible to create important partnerships in order to guide the work of teachers, as they will be the role models for students aged 6 to 12.

In relation to the academic aspects, eight out of ten have an average mark of “notable”, and they entered university through the baccalaureate, more than half of the sample receiving a scholarship to study at the university. These data are consistent with the University Indicator Statistics (2018). Results were obtained with an average score of 7.22 points out of 10, which was related to the highest percentage of individuals who received the university scholarship. Similarly, taking into account the previous training that gave access to university studies in our participants, it coincides with the notes collected by Lorenzo et al. [44] as well as by Martínez et al. [2], with higher percentages of high school students who access higher studies pointing to over-qualification, and considering professional training as the option most suitable for the employment opportunities and job insertion.

With regard to self-concept, high scores were obtained in all dimensions except for emotional self-concept, which showed a half point difference from the others. In this sense, Montoya, Pinilla and Dussán [45] note that this could be due to the situation experienced by university students, as they face a period of high demands in terms of training, as well as the confrontation of social expectations regarding professional life and labour insertion.

Alcohol and tobacco are not substances consumed in four out of five of the participants in the study, with around four out of twenty-five having a low risk with alcohol consumption and medium dependency on tobacco. According to the latest Survey on Alcohol and Drugs in Spain (OEDA) [46], the prevalence of alcohol and tobacco consumption is presented as the most consumed substances, followed by hypnosedants and cannabis, establishing as pattern that those with risk consumption of alcohol are more likely to be exposed to situations related to the consumption of other drugs.

With regard to emotional intelligence, it is established that those who access university through professional training have higher average levels than those who enter through a bachelor’s degree, despite only finding significance in the dimension of self-emotional management, these results are observed in the other dimensions. Emotional intelligence is defined as the condition that a person has when interacting with different stimulations, states or situations in their environment [47]. In the educational field, the need to promote emotional training and capacities for self-control, empathy, communication, inter-relationship, among others, is considered, not only with emphasis on disciplinary and theoretical knowledge [48,49]. In this sense, the results could be adjusted to the approach of Ministry of Education and Science Order 78/2010 [50] of 27 August, which states that training programmes prepare students for activity within a professional field, enabling them not only to adjust themselves to changes in the productive system, but also to contribute to their professional, personal and social development [51].

The average mark in exams is related to self-concept and emotional intelligence, since those with marks of note present higher levels of self-concept in general and in their academic dimension, as well as the dimensions of hetero-emotional management and emotional use, while those with marks of pass obtain better levels of physical self-concept than those who obtain excellent. These data are related to those provided by Calet and Dumitrache [52], Martínez-Martínez and González-Hernández [53] or the relationship of positive self-concept and better levels of emotional intelligence with academic performance, as well as academic success, contributes to the students’ self-concept [54]. In this regard, the self-concept is related to motivation and learning strategies, whereas a positive self-concept encourages deeper processing strategies for study and support; while a negative self-concept is related to the practice of more superficial learning strategies that lead to less academic success [55,56]. Similarly, Fernández-Lasarte, Ramos-Díaz and Axpe-Sáez [57] state that the contribution of emotional intelligence to good academic performance could be due, for example, to the ability to regulate emotions appropriately in order to reduce negative emotions, failures and stress, among others, which damage intellectual activity.

Similarly, academic marks are significantly related to tobacco consumption, where those with excellent marks are non-smokers overall. While those with passing and notable marks represent approximately one out of every five smokers with medium dependency. The consumption of harmful substances to health is negatively related to implicit factors in the educational environment, as it is evident in some studies whether these substances may harm the academic performance of young people [58,59]. On the contrary, there is also research that has not found relationships between academic variables and the consumption of harmful substances [60,61]; this may be motivated by establishing the frequency and duration of substance consumption, understanding prolonged consumption as the one that carries negative or more disturbing consequences for health.

In the same direction, students who receive a scholarship are those who have significantly higher levels of emotional intelligence, in the dimension of emotional perception, compared to those who do not receive it. As several studies have pointed out, the academic requirements for obtaining a scholarship have positive effects on the level of education achieved, since the scholarship provides students an incentive to persist, make an effort and commit to their education; it facilitates an academic process that is less linked to economic conditions; they reduce the need to work to pay university fees and costs and the feelings of responsibility and guilt towards families for this issue [62,63,64]. This issue could be related to high levels of emotional intelligence, since the existence of this positive stimulation bringing well-being to students will reinforce emotional skills throughout the process.

In terms of self-concept and the acquisition of scholarships by university students, those who receive them are the ones who have the highest levels of general, academic and physical self-concept, while those who do not have it show a higher level of family self-concept. As with emotional intelligence, those students who receive a scholarship have a positive motivation to undertake different strategies and face different situations more successfully, unlike those who do not receive this help. The difference in the family dimension can be explained by the fact that the possibility of carrying out university studies without a grant requires economic support mainly from families and, hence, the perception of family support and assistance for those who do not receive a grant.

## 5. Conclusions

Finally, the main conclusions are based on the fact that the levels of general self-concept and self-emotional management are higher in young people who access university through professional training, as well as in those who are rated as excellent in terms of the academic dimension of self-concept and hetero-emotional management, while physical self-concept and emotional use improve in those with lower grades. Receiving a scholarship to study points to higher levels in most of the dimensions of emotional intelligence and self-concept for those who receive this support. Finally, harmful substances did not show significant relationships with academic factors, except between tobacco and the average mark. Nevertheless, it should be pointed out that this study presents limitations such as the type of methodological design used, because it does not enable cause–effect conclusions to be drawn, as well as variables that do not understand certain psychological aspects or factors that enable the situation involving our study sample to be known. Regarding prospective studies, it would be interesting to carry out longitudinal studies that enable the inclusion of the motivational parameters, stress or burnout levels and life satisfaction, among others, within a broader and clearer perspective.

The present study was not free of limitations, as it is a descriptive and cross-sectional study design with the isolated measurement of a single group. Thus, one of the virtues of this study is that it was carried out on future physical education teachers, which does not allow for the generalisation of the whole group of teachers as could be specialities such as primary or pre-school education among others. It would be interesting for future studies to address this line of research from different points of measurement, in order to establish cause–effect relationships. As it cannot be generalised to different groups, another line of research that is opening up is to establish a comparison between different educational specialists. In this way, this study can act as a basis for educational centres and state governments, in order to create measures and establish intervention programmes between students and teachers.

## Figures and Tables

**Table 1:** Descriptive Analysis.

**Sex**		**Self-Concept M (SD)**		**Tobacco Consumption**	
Men	58.7% (*n* = 455)	General self-concept	3.58 (0.31)	Non-smoker	81.7% (*n* = 633)
Women	41.3% (*n* = 320)	Academic self-concept	3.76 (0.52)	Low dependency	0.9% (*n* = 7)
**Average Mark**		Social self-concept	3.54 (0.36)	Medium dependency	15.5% (*n* = 120)
Excellent	2.1% (*n* = 16)	Emotional self-concept	3.26 (0.78)	High dependency	1.9% (*n* = 15)
Notable	82.3% (*n* = 638)	Family self-concept	3.56 (0.33)		
Pass	15.6% (*n* = 121)	Physical self-concept	3.77 (0.62)		
**University Access**				**Emotional Intelligence M (SD)**	
Professional training	11.1% (*n* = 86)	**Alcohol Consumption**		General emotional intelligence	3.95 (0.43)
Baccalaureate	88.9% (*n* = 689)	Risk-free drinker	82.2% (*n* = 637)	Emotional perception	4.11 (0.50)
**Getting Scholarship**		Low-risk drinker	16.3% (*n* = 126)	Self-emotional management	3.73 (0.52)
Yes	58.7% (*n* = 463)	Medium-risk drinker	1.0% (*n* = 8)	Hetero-emotional management	3.99 (0.48)
No	40.3% (*n* = 312)	High-risk drinker	0.5% (*n* = 4)	Emotional use	3.96 (0.56)

**Table 2:** Psychosocial factors according to university access.

Variable		University Access		*Sig.*
		Baccalaureate M (SD)	Professional Training M (SD)	
**Self-concept**	General	3.57 (0.304)	3.64 (0.310)	0.041 *
	Academic	3.75 (0.524)	3.81 (0.493)	0.362
	Social	3.53 (0.362)	3.61 (0.374)	0.052
	Emotional	3.25 (0.773)	3.35 (0.829)	0.247
	Family	3.56 (0.332)	3.60 (0.279)	0.261
	Physical	3.77 (0.614)	3.84 (0.643)	0.277
**Alcohol consumption**	No risk	89.2% (*n* = 568)	10.8% (*n* = 69)	0.556
	Low risk	87.3% (*n* = 110)	12.7% (*n* = 16)	
	Medium risk	87.5% (*n* = 7)	12.5% (*n* = 1)	
	High risk	100.0% (*n* = 4)	0% (*n* = 0)	
**Tobacco consumption**	Non-smoking	88.3% (*n* = 559)	11.7% (*n* = 74)	0.551
	Low dependency	100% (*n* = 7)	0% (*n* = 0)	
	Medium dependency	91.7% (*n* = 110)	8.3% (*n* = 10)	
	High dependency	86.7% (*n* = 13)	13.3% (*n* = 2)	
**Emotional intelligence**	General	3.94 (0.415)	4.04 (0.548)	0.088
	Emotional perception	4.10 (0.490)	4.16 (0.593)	0.341
	Self-emotional management	3.71 (0.514)	3.88 (0.564)	0.004 *
	Hetero-emotional management	3.98 (0.468)	4.08 (0.603)	0.171
	Emotional use	3.95 (0.554)	4.04 (0.649)	0.212

Note 1. Statistically significant differences at level *p* < 0.05 (*).

**Table 3:** Psychosocial factors according to average mark.

Variable		Average Mark			*Sig.*
		Pass (N = 121) M (SD)	Notable (N = 638) M (SD)	Excellent (N = 16) M (SD)	
**Self-concept**	General	3.48 (0.262)	3.60 (0.308)	3.53 (0.336)	*p* ≤ 0.05 ^a^
	Academic	3.43 (0.537)	3.81 (0.491)	4.07 (0.611)	*p* ≤ 0.05 ^a,b^
	Social	3.53 (0.417)	3.54 (0.356)	3.47 (0.250)	*p* ≥ 0.05
	Emotional	3.22 (0.768)	3.27 (0.786)	3.21 (0.628)	*p* ≥ 0.05
	Family	3.56 (0.333)	3.56 (0.327)	3.52 (0.264)	*p* ≥ 0.05
	Physical	3.63 (0.552)	3.81 (0.621)	3.40 (0.704)	*p* ≤ 0.05 ^a,c^
**Alcohol consumption**	No risk	15.2% (*n* = 97)	82.4% (*n* = 525)	2.4% (*n* = 15)	0.556
	Low risk	0.19 (*n* = 24)	80.2% (*n* = 101)	0.8% (*n* = 1)	
	Medium risk	0% (*n* = 0)	100% (*n* = 8)	0% (*n* = 0)	
	High risk	0% (*n* = 0)	100.0% (*n* = 4)	0% (*n* = 0)	
**Tobacco consumption**	Non-smoking	14.1% (*n* = 89)	83.4% (*n* = 528)	2.5% (*n* = 16)	0.013 *
	Low dependency	42.9% (*n* = 3)	57.1% (*n* = 4)	0% (*n* = 0)	
	Medium dependency	19.2% (*n* = 23)	80.8% (*n* = 97)	0% (*n* = 0)	
	High dependency	40.0% (*n* = 6)	60.0% (*n* = 9)	0% (*n* = 0)	
**Emotional intelligence**	General	3.88 (0.466)	3.96 (0.427)	3.85 (0.361)	0.101
	Emotional perception	4.03 (0.523)	4.12 (0.500)	4.10 (0.376)	0.162
	Self-emotional management	3.73 (0.511)	3.73 (0.525)	3.54 (0.484)	0.354
	Hetero-emotional management	3.89 (0.565)	4.01 (0.469)	3.93 (0.417)	0.039 *
	Emotional use	3.86 (0.573)	3.98 (0.565)	3.76 (0.451)	0.036 *

Note 1. Statistically significant differences at level *p* < 0.05 (*). Note 2. Group differences between pass and notable (a); group differences between pass and excellent (b); group differences between notable and excellent (c).

**Table 4:** Psychosocial factors according to getting scholarship.

Variable		Getting Scholarship		*Sig.*
		Yes M (SD)	No M (SD)	
**Self-concept**	General	3.60 (0.303)	3.55 (0.306)	0.045 *
	Academic	3.82 (0.502)	3.67 (0.535)	0.000 *
	Social	3.53 (0.378)	3.55 (0.343)	0.468
	Emotional	3.28 (0.799)	3.23 (0.750)	0.411
	Family	3.53 (0.338)	3.61 (0.303)	0.001 *
	Physical	3.82 (0.581)	3.70 (0.663)	0.009 *
**Alcohol consumption**	No risk	60.9% (*n* = 388)	39.1% (*n* = 249)	0.065
	Low risk	56.3% (*n* = 71)	43.7% (*n* = 55)	
	Medium risk	50.0% (*n* = 4)	50.0% (*n* = 4)	
	High risk	0% (*n* = 0)	100% (*n* = 4)	
**Tobacco consumption**	Non-smoking	61.3% (*n* = 388)	38.7% (*n* = 245)	0.178
	Low dependency	57.1% (*n* = 4)	42.9% (*n* = 3)	
	Medium dependency	50.8% (*n* = 61)	49.2% (*n* = 59)	
	High dependency	66.7% (*n* = 10)	33.3% (*n* = 5)	
**Emotional intelligence**	General	3.96 (0.448)	3.93 (0.409)	0.334
	Emotional perception	4.14 (0.495)	4.06 (0.510)	0.042 *
	Self-emotional management	3.72 (0.540)	3.74 (0.495)	0.650
	Hetero-emotional management	4.01 (0.489)	3.97 (0.480)	0.212
	Emotional use	3.96 (0.578)	3.96 (0.548)	0.920

Note 1. Statistically significant differences at level *p* < 0.05 (*).

## Data Availability

Data sharing not applicable.
